# Basal and Ischemia-Induced Transcardiac Troponin Release into the Coronary Circulation in Patients with Suspected Coronary Artery Disease

**DOI:** 10.1371/journal.pone.0060163

**Published:** 2013-04-02

**Authors:** Masaaki Konishi, Seigo Sugiyama, Koichi Sugamura, Toshimitsu Nozaki, Keisuke Ohba, Junichi Matsubara, Kenji Sakamoto, Yasuhiro Nagayoshi, Hitoshi Sumida, Eiichi Akiyama, Yasushi Matsuzawa, Kentaro Sakamaki, Satoshi Morita, Kazuo Kimura, Satoshi Umemura, Hisao Ogawa

**Affiliations:** 1 Departments of Cardiovascular Medicine, Faculty of Life Sciences, Graduate School of Medical Sciences, Kumamoto University, Kumamoto, Japan; 2 Division of Cardiology, Yokohama City University Medical Center, Yokohama, Japan; 3 Department of Biostatistics and Epidemiology, Yokohama City University Medical Center, Yokohama, Japan; 4 Department of Medical Science and Cardiorenal Medicine, Yokohama City University Graduate School of Medicine, Yokohama, Japan; S.G.Battista Hospital, Italy

## Abstract

**Background:**

Cardiac troponin is a specific biomarker for cardiomyocyte necrosis in acute coronary syndromes. Troponin release from the coronary circulation remains to be determined because of the lower sensitivity of the conventional assay. We sought to determine basal and angina-induced troponin release using a highly sensitive troponin assay.

**Methods and Results:**

The cardiac troponin T levels in serum sampled from the peripheral vein (PV), the aortic root (AO), and the coronary sinus (CS) were measured in 105 consecutive stable patients with coronary risk factor(s) and suspected coronary artery disease (CAD) and in 33 patients without CAD who underwent an acetylcholine provocation test. At baseline, there was a significant increase in the troponin levels from AO [9.0 (6.4, 13.1) pg/mL for median (25^th^, 75^th^ percentiles)] to CS [10.3 (7.3, 15.5) pg/mL, p<0.001] in 96 (91.4%) patients and the difference was 1.1 (0.4, 2.1) pg/mL, which reflected basal transcardiac troponin release (TTR). TTR was positively correlated with PV levels (r = 0.22, p = 0.03). Male sex, left ventricular hypertrophy determined by echocardiography, T-wave inversion, and CAD correlated with elevated TTR defined as above: median, 1.1 pg/mL. A significant increase in TTR was noted in 17 patients with coronary spasms [0.6 (0.2, 1.2) pg/mL, p<0.01] but not in 16 patients without spasms [0.0 (−0.5, 0.9) pg/mL, p = 0.73] after the acetylcholine provocation.

**Conclusion:**

Basal TTR in the coronary circulation was observed in most of the patients with suspected CAD and risk factor(s). This sensitive assay detected myocardial ischemia-induced increases in TTR caused by coronary spasms.

## Introduction

Cardiac troponin is well established as a specific biomarker for cardiomyocyte necrosis in individuals with acute coronary syndromes (ACS) but not in individuals with angina pectoris [1]. The latest generation of sensitive troponin assays has the improved sensitivity for the diagnosis of myocardial infarction but the specificity is reduced as compared with the standard assays [2]. The elevation of troponin levels is often observed in non-ACS settings [3] and may partially account for the reduced specificity with respect to the diagnosis of ACS. However, the clinical and pathophysiological significance of minimally increased levels of troponin remains controversial [4]. The level of troponin release from the coronary circulation in stable physical states and during transient myocardial ischemia due to angina has not been determined because of the low sensitivity of the conventional troponin assay. We assessed the basal and angina-induced occurrence of troponin release using a highly sensitive troponin T assay in stable states and before and after acetylcholine (ACh)-induced coronary spasm.

## Methods

The present study was approved by the Ethics Review Committee of Kumamoto University (Kumamoto, Japan). Signed informed consent was obtained from each patient before participation. This study is registered at the UMIN protocol registration system (UMIN000005099). This study was conducted in accordance with the ethical principles originating in the Declaration of Helsinki.

### 

#### Clinical trial registration

https://center.umin.ac.jp ID: UMIN000005099.

### Study Population

The cardiac troponin T levels in serum sampled from the peripheral vein, the aortic root, and the coronary sinus were measured using a highly sensitive assay in 105 consecutive stable patients who had at least 1 coronary risk factor, were suspected of having coronary artery disease (CAD), and underwent coronary angiography from April 2008 to September 2009. In the same period, samples collected before and after the ACh provocation test were also assessed in 33 patients without CAD.

### Coronary Angiography and the ACh Test

A 6-F catheter was placed in the coronary sinus to sample blood during coronary angiography. CAD was defined to be ≥75% stenosis (according to the classification set by the American Heart Association) on conventional coronary angiography analyzed quantitatively by software (CAAS; Pie Medical Imaging, Maastricht, The Netherlands). The ACh test was indicated for patients with chest discomfort at rest in the absence of CAD. Patients with heart failure were excluded from the ACh test. Incremental doses (20, 50, and 100 µg) of ACh were injected into the left coronary artery, and angiography was performed 1 min after each injection. Then, 50 µg of ACh was injected into the right coronary artery. At baseline and either ACh-induced coronary spasm or 1 min after injection of the maximum dose of ACh, paired samples were collected simultaneously from the aortic root and coronary sinus. This method is described in the current guidelines of vasospastic angina [5] as an assessment of myocardial lactate consumption. Coronary spasm was defined as >90% lumen narrowing of the epicardial coronary artery according to this guideline [5].

### Measurement of Levels of Highly Sensitive Troponin T

Blood was processed immediately and frozen at 80°C until it was assayed by a commercial highly sensitive troponin T assay method (Roche Diagnostics, Penzberg, Germany). Because of the enhanced sensitivity, this assay is reported to have a coefficient of variation of 8% at 10 pg/mL [6] and 99th percentile for a normal reference population is reported to be 13 pg/mL [7].

### Assessment of Risk Factors and Covariates

Blood was drawn after an overnight fast. Diabetes mellitus (DM) was diagnosed based on criteria set by the World Health Organization or the use of hypoglycemic agents or insulin. Hypertension was defined as a systolic blood pressure ≥140 mmHg, a diastolic blood pressure ≥90 mmHg, or the use of an antihypertensive treatment. The estimated glomerular filtration rate was calculated using a modified formula from the Modification of Diet in Renal Disease study equation, which was proposed by the Japanese Society of Nephrology [8]. The ten-year coronary heart disease risk was calculated using the Framingham risk score algorithm [9]. Measurement of left ventricular ejection fraction was done in biplane apical (two- and four-chamber) views using a modified version of Simpson’s method in echocardiography. Left ventricular hypertrophy was defined as an increase in left ventricular mass index >149 g/m^2^ for men and >122 g/m^2^ for women in echocardiography [10] and by Sokolow–Lyon voltage criteria in electrocardiography [11]. T-wave inversion was assessed in lead I, II, aV_L_, and V_3_–V_6_ [12]. Heart failure was defined as a functional capacity of class II or III as set by the New York Heart Association [13].

### Statistical Analyses

Data are expressed as mean ± standard deviation or median and 25^th^–75^th^ percentiles, as appropriate. Differences in troponin T levels between the aortic root and the coronary sinus were analyzed by the Wilcoxon rank sum test. Pearson correlations between troponin T in peripheral veins and transcardiac troponin release among patients with significant transcardiac release (N = 91) were analyzed after log-transformation of each variable. Differences in transcardiac troponin release between two groups with respect to coronary risk factors and patient diseases were analyzed by the Mann–Whitney *U* test. Univariate and multivariate logistic regression analyses for higher basal transcardiac troponin release defined as above: median, 1.1 pg/mL, were undertaken among certain risk factors. Bootstrapping was used for esvaluation of the internal validity of the variable selection in the multivariate models. We also calculated the Hosmer–Lemeshow goodness-of-fit statistic. *P*<0.05 denoted statistical significance, and all tests were two-tailed. Variables were log-transformed if they had a skewed distribution. All analyses were undertaken using SPSS 17.0J for Windows (SPSS Inc., Tokyo, Japan) and SAS software, version 9.3 (SAS Institute Inc., Cary, NC, USA).

## Results

### Serum Cardiac Troponin Levels Sampled from the Peripheral Vein, the Aortic Root, and the Coronary Sinus

**Figure 1:**
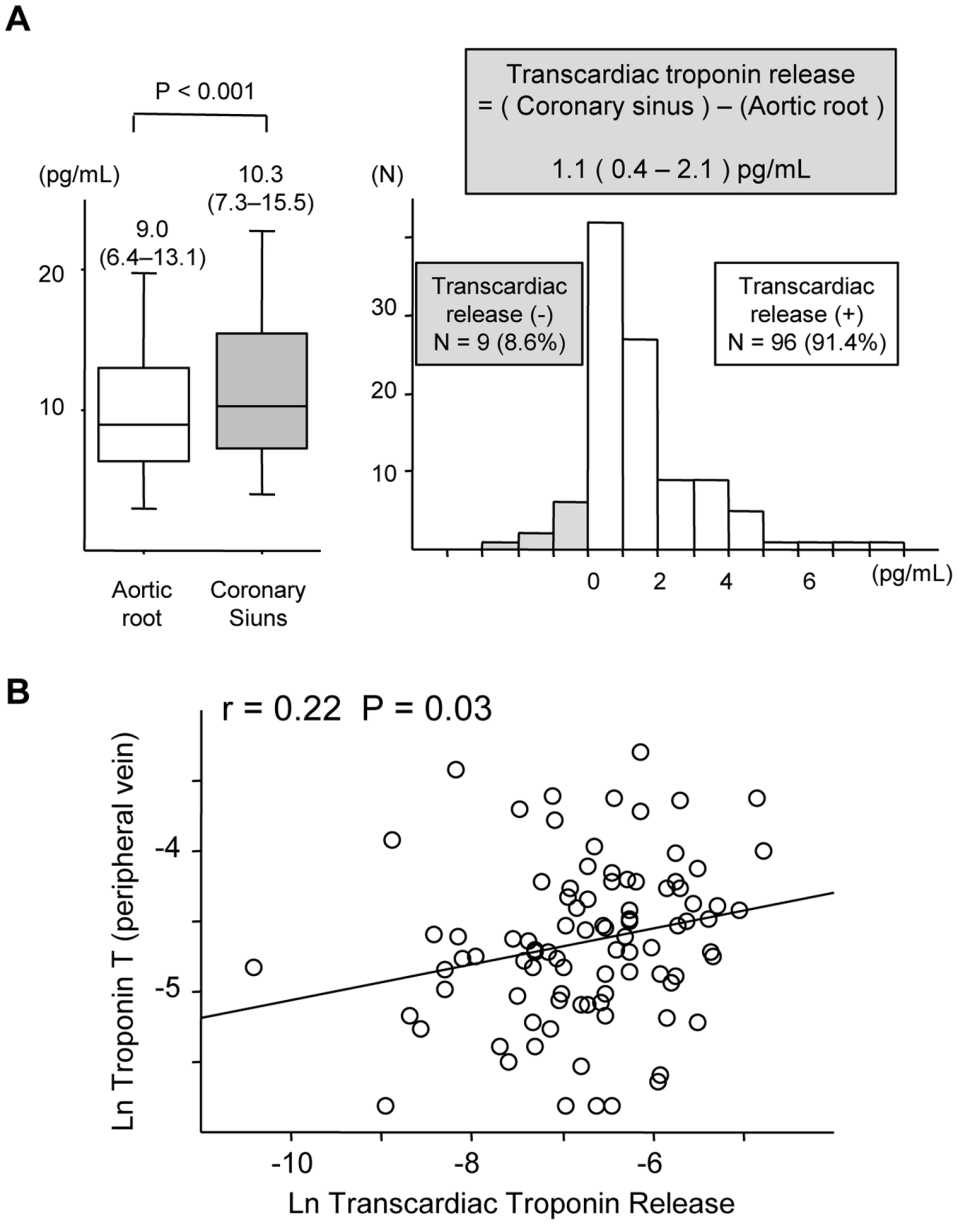
Levels of troponin T in the aortic root, coronary sinus, and peripheral vein. (A) The serum troponin T levels in the aortic root and coronary sinus and the distribution of transcardiac troponin release in patients in stable physical states. There was a significant increase in the troponin T in the coronary sinus compared to the aortic root in 91.4% of patients. The difference in the troponin T level between the coronary sinus and the aortic-root level was 1.1 (0.4, 2.1) pg/mL, which reflects basal transcardiac troponin release. (B) The correlation between transcardiac troponin release and troponin T in peripheral veins. Each variable is log-transformed. Transcardiac troponin release was positively correlated with peripheral-vein levels (r = 0.22, p = 0.03).

**Figure 2:**
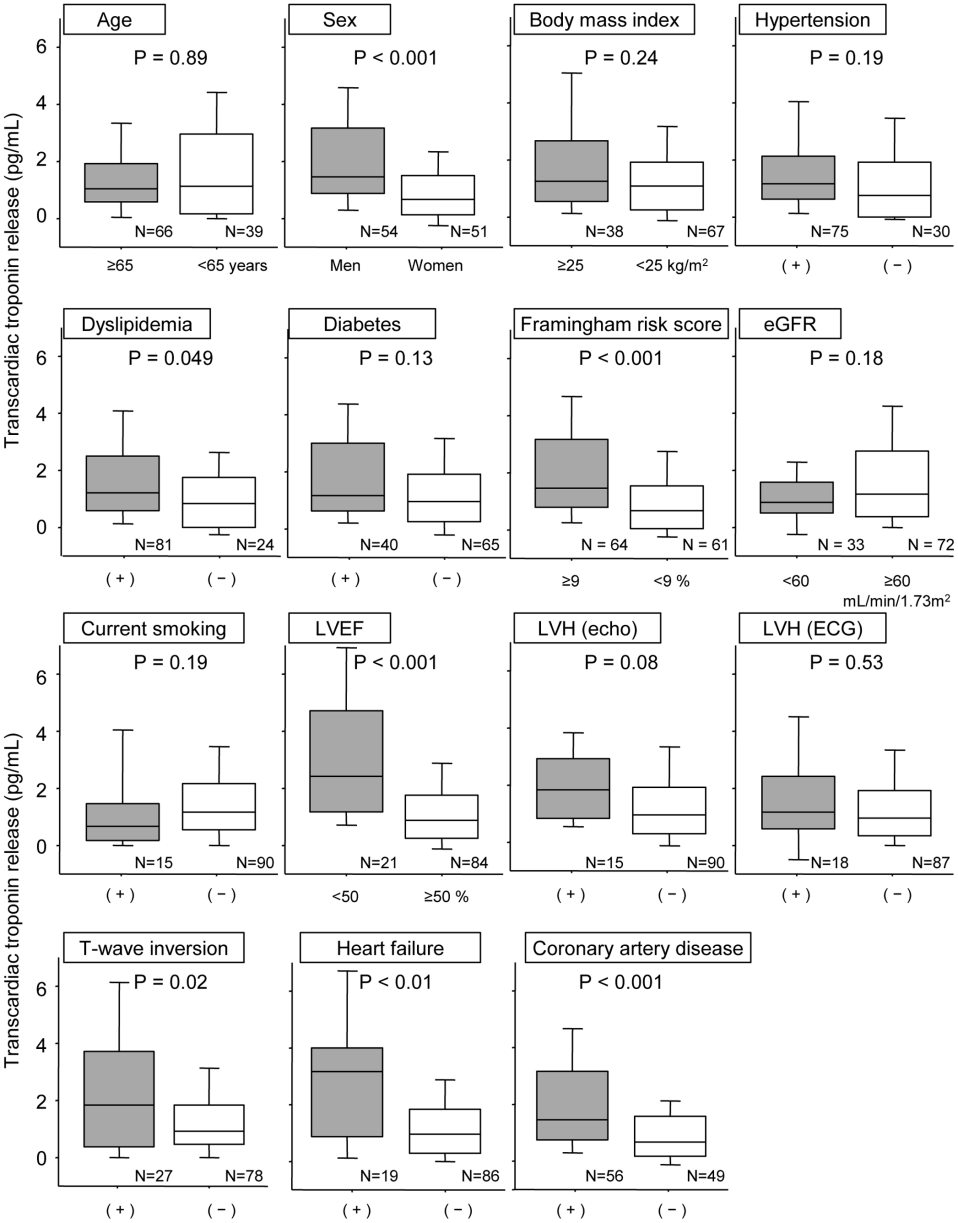
Difference in transcardiac troponin release among subgroups. Transcardiac troponin release was significantly higher in patients with coronary artery disease (CAD) than in patients without CAD (p<0.001). Transcardiac troponin release was also significantly higher in patients with heart failure compared with those without heart failure (p<0.01). Transcardiac troponin release was higher in men and in patients with a higher Framingham risk scores or negative T waves.

Table 1 shows the characteristics of the study subjects (n = 105). All subjects underwent conventional coronary angiography. Fifty-six patients were found to have significant organic stenosis in major coronary arteries. Troponin T in peripheral veins was detectable in 95 (90.5%) patients, and the median value was 9.6 pg/mL. The levels of troponin T in the aortic root and coronary sinus were also measured. At baseline, there was a significant increase in the troponin T levels in the coronary sinus [median (25^th^, 75^th^ percentile) = 10.3 (7.3, 15.5) pg/mL] compared to the aortic root [9.0 (6.4, 13.1) pg/mL, p<0.001 ] in 96 (91.4%) patients. The difference in the troponin T levels between the coronary sinus and the aortic-root level was 1.1 (0.4, 2.1) pg/mL; the difference reflected basal transcardiac troponin release (Figure 1A). Transcardiac troponin release was positively correlated with peripheral-vein levels of troponin (r = 0.22, p = 0.03: Figure 1B) after log-transformation. The transcardiac troponin release was significantly higher in men, and in patients with dyslipidemia, higher Framingham risk scores (defined as above median (9%)), lower left ventricular ejection fraction (defined as <50%), negative T waves, heart failure (16 patients (84%) were New York Heart Association class II and 3 patients (16%) class III), or CAD. Using a transcardiac troponin release cutoff value of >1.1 pg/mL (median), the sensitivity, specificity, positive predictive value and negative predictive value for the detection of CAD were 57%, 69%, 68%, and 59%, respectively. In patients with CAD, however, the transcardiac troponin release was comparable between patients with single-vessel (n = 20) and multi-vessel (n = 36) disease (p = 0.95). The transcardiac troponin release was comparable between patients regardless of age (above/equal to or below 65 years), body mass index (BMI; above/equal to or below 25 kg/m^2^), hypertension, DM, lower estimated glomerular filtration rate (above/equal to or below 60 mL/min/1.73 m^2^), current smoking status, and left ventricular hypertrophy (as determined by echocardiography and electrocardiography) (Figure 2). We assessed factors associated with elevated transcardiac troponin release (defined as above: median, 1.1 pg/mL) with logistic regression analysis. Male sex (odds ratio [OR]: 4.15, 95% confidence interval [CI]: 1.82–9.46, p<0.001), the presence of T-wave inversion (OR: 3.38, 95% CI: 1.34–8.50, p<0.01), heart failure (OR: 3.31, 95% CI: 1.15–9.56, p = 0.03), and CAD (OR: 3.02, 95% CI: 1.35–6.77, p<0.01) were revealed to have significant relationships with elevated transcardiac troponin release. Multivariate logistic regression analyses revealed that male sex (OR: 11.78, 95% CI: 3.23–42.99, p<0.001), left ventricular hypertrophy by echocardiography (OR: 5.55, 95% CI: 1.29–23.88, p = 0.02), T-wave inversion (OR: 8.29, 95% CI: 2.33–29.44, p<0.01), and CAD (OR: 2.90, 95% CI: 1.03–8.17, p = 0.04) were independently associated with elevated transcardiac troponin release (Table 2). The Hosmer–Lemeshow goodness-of-fit statistic was 0.25 and this model was well-calibrated.

**Table 1:** Patient characteristics.

Characteristic	Total
Number	105
Age, years	67(11)
Male sex, n (%)	54 (51)
Body mass index, kg/m^2^	24.2 (3.5)
Hypertension, n (%)	77 (72)
Systolic blood pressure, mmHg	126 (19)
Diastolic blood pressure, mmHg	73 (13)
Dyslipidemia, n (%)	81 (77)
Total cholesterol, mg/dL	180 (37)
LDL cholesterol, mg/dL	103 (32)
HDL cholesterol, mg/dL	53 (15)
Triglycerides, mg/dL*	104 (82–139)
Diabetes mellitus, n (%)	40 (38)
Fasting plasma glucose, mg/dL	98 (18)
Hemoglobin A1c, %*	5.9 (5.6–6.5)
Framingham risk score, %	12 (9)
eGFR, mL/min/1.73 m^2^	73 (22)
Current smoking, n (%)	15 (14)
LVEF, %	59 (12)
Left ventricular mass index, g/m^2^	104 (31)
Left ventricular hypertrophy (echo), n (%)	15 (14)
Left ventricular hypertrophy (ECG), n (%)	18 (17)
T-wave inversion, n (%)	27 (26)
Heart failure, n(%)	19 (18)
Coronary artery disease, n (%)	56 (53)
Medications	
Aspirin, n (%)	65 (62)
Statins, n (%)	52 (50)
ACE inhibitors/ARBs, n (%)	51 (49)

Data are the mean (standard deviation) or number (percentage).

* Median and 25th–75th percentiles. LDL: low-density lipoprotein, HDL: high-density lipoprotein, eGFR: estimated glomerular filtration rate, LVEF: left ventricular ejection fraction, ACE: angiotensin converting enzyme, ARB: angiotensin II receptor blocker.

**Table 2:** Univariate and multivariate logistic regression analyses of possible factors associated for higher basal transcardiac troponin release (above median: 1.1 pg/mL).

	Univariate		Multivariate	
Factor	OR (95%CI)	p	OR (95%CI)	p
Age (per year)	1.00 (0.97–1.04)	0.95	Not selected	
Male sex	4.15 (1.82–9.46)	<0.001	11.78 (3.23–42.99)	<0.001
Body mass index (per 1 kg/m^2^)	1.03 (0.92–1.15)	0.64	Not selected	
Hypertension	1.31 (0.56–3.10)	0.54	Not selected	
Dyslipidemia	1.86 (0.72–4.82)	0.20	Not selected	
Diabetes mellitus	1.02 (0.46–2.24)	0.97	Not selected	
Framingham risk score (per 1%)	1.01 (0.97–10.6)	0.52	0.95 (0.89–1.01)	0.08
eGFR (per 10 ml/min/1.73 m^2^)	1.02 (0.86–1.22)	0.34	Not selected	
Current smoking	0.57 (0.18–1.81)	0.34	0.25 (0.06–1.11)	0.07
LVEF (per 10%)	0.72 (0.52–1.00)	0.05	Not selected	
LVH (echo)	2.05 (0.67–6.26)	0.21	5.55 (1.29–23.88)	0.02
LVH (ECG)	0.99 (0.36–2.73)	0.98	0.25 (0.06–1.04)	0.06
T- wave inversion	3.38 (1.34–8.50)	<0.01	8.29 (2.33–29.44)	<0.01
Heart failure	3.31 (1.15–9.56)	0.03	Not selected	
Coronary artery disease	3.02 (1.35–6.77)	<0.01	2.90 (1.03–8.17)	0.04

eGFR: estimated glomerular filtration rate, LVEF: left ventricular ejection fraction, LVH: left ventricular hypertrophy.

### Transcardiac Troponin Release Before and After the ACh Test

**Figure 3:**
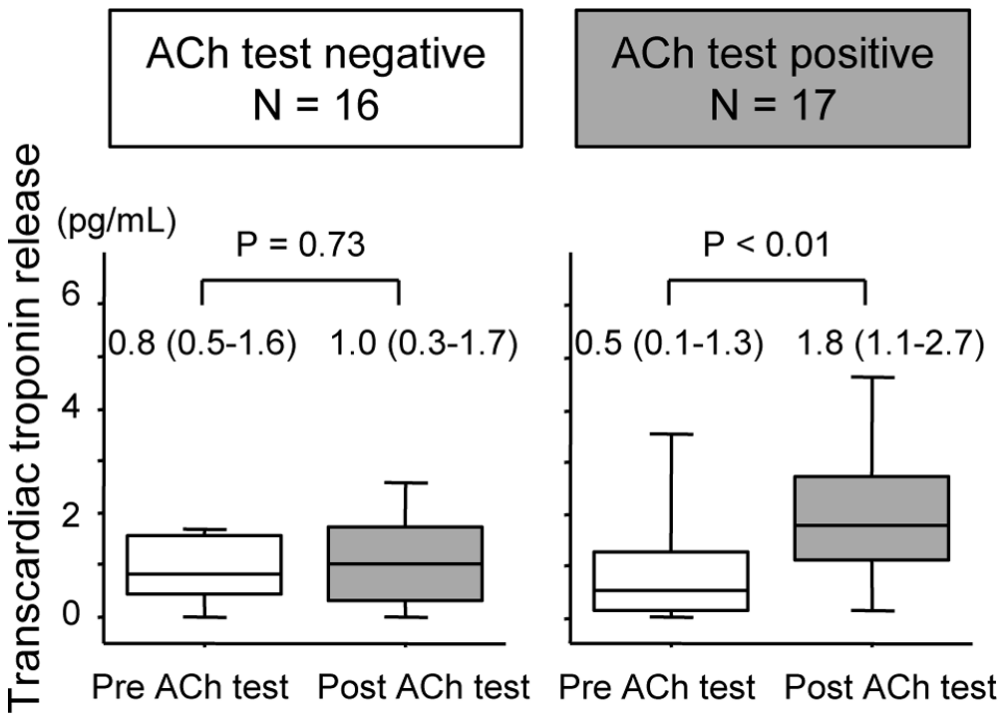
Transcardiac troponin release before and after the ACh test. There was a significant increase in transcardiac troponin release after the ACh provocation test in 17 patients with coronary spasms (p<0.01 for the comparison with baseline) but not in 16 patients without spasms (p = 0.73).

Transcardiac troponin release was also assessed before and after provocation in patients without CAD who underwent the ACh test (N = 33). Table 3 shows the characteristics of the participating patients with positive or negative results of the ACh test. Positive ACh results were accompanied by chest pain in all patients. Age, sex, BMI, coronary risk factors, and medications were comparable between the two groups. The transcardiac troponin release before the ACh test was 0.5 (0.1, 1.3) pg/mL in 17 patients with positive results and 0.8 (0.5, 1.6) in 16 patients with negative results (p = 0.53, Table 4). In patients with a positive ACh test, 13 subjects (76%) showed an increase in the transcardiac troponin release as the result of the ACh test, whereas 3 (19%) patients with negative result showed an increase in the transcardiac troponin release (p = 0.04). There was a significant increase in the transcardiac troponin release after the ACh provocation test in patients with coronary spasms [0.6 (0.2, 1.2) pg/mL, p<0.01 for the comparison with baseline] but not in patients without spasms [0.0 (−0.5, 0.9) pg/mL, p = 0.73] (Table 4, Figure 3).

**Table 3:** Characteristics of patients who underwent the ACh test.

Characteristics	Total	ACh positive	ACh negative	P
Number	33	17	16	
Age, years	59 (13)	59 (13)	60 (14)	0.83
Male sex, n (%)	14 (42)	5 (29)	9 (56)	0.17
Body mass index, kg/m^2^	23.4 (3.9)	23.5 (4.5)	23.3 (3.4)	0.87
Hypertension, n (%)	14 (42)	6 (35)	8 (50)	0.49
Systolic blood pressure, mmHg	120 (17)	119 (15)	122 (19)	0.66
Diastolic blood pressure, mmHg	69 (9.2)	69 (8)	70 (11)	0.61
Dyslipidemia, n (%)	21 (64)	10 (59)	11 (69)	0.72
Total cholesterol, mg/dL	184 (32)	177 (26)	192 (36)	0.17
LDL cholesterol, mg/dL	109 (31)	102 (23)	117 (38)	0.18
HDL cholesterol, mg/dL	54 (13)	54 (12)	53 (14)	0.91
Triglycerides, mg/dL*	122 (87–158)	108 (83–170)	127 (95–149)	0.77
Diabetes mellitus, n (%)	2 (6)	1 (6)	1 (6)	1.00
Fasting plasma glucose, mg/dL	93 (16)	90 (10)	97 (21)	0.23
Hemoglobin A1c, %*	5.7 (5.4–6.0)	5.7 (5.3–5.8)	5.7 (5.5–6.3)	0.25
Framingham risk score, %	8 (6)	6 (4)	10 (7)	0.08
eGFR, ml/min/1.73 m^2^	84 (23)	82 (24)	85 (21)	0.77
Current smoking, n (%)	12 (36)	6 (35)	6 (38)	1.00
Medications				
Aspirin, n (%)	14 (42)	10 (59)	4 (25)	0.08
Statins, n (%)	8 (24)	5 (29)	3 (19)	0.69
ACE inhibitors/ARBs, n (%)	9 (27)	6 (35)	3 (19)	0.44

Data are the mean (standard deviation) or number (percentage).

* Median and 25th–75th percentiles. LDL: low-density lipoprotein, HDL: high-density lipoprotein, eGFR: estimated glomerular filtration rate, ACE: angiotensin converting enzyme, ARB: angiotensin II receptor blocker.

**Table 4:** Transcardiac troponin release of patients with positive or negative ACh tests.

	Total	ACh-positive	ACh-negative	p
Number	33	17	16	
Transcardiac troponin release, pg/mL				
Baseline	0.8 (0.2–1.4)	0.5 (0.1–1.3)	0.8 (0.5–1.6)	0.53
After the ACh test	1.4 (0.6–2.2)	1.8 (1.1–2.7)*	1.0 (0.3–1.7)†	0.10
ΔTranscardiac troponin release ([After ACh test] to [Baseline])	0.5 (–0.1–1.1)	0.6 (0.2–1.2)	0.0 (–0.5–0.9)	0.049
Patients with an increase in transcardiac troponin release, n (%)	19 (58)	13 (76)	3 (19)	0.04

Data are the median (25th–75th percentiles) or number (percentage).

* p<0.01 for the comparison with baseline.

† p = 0.73 for the comparison with baseline.

## Discussion

Basal transcardiac troponin release in the coronary circulation was observed in most of the patients with suspected CAD and coronary risk factor(s). Male sex, left ventricular hypertrophy determined by echocardiography, T-wave inversion, and CAD correlated with elevated troponin release under stable physical conditions. This sensitive assay detected significant angina-like transient myocardial ischemia-induced significant increase in transcardiac troponin release in patients with coronary spasms in the absence of CAD.

### Transcardiac Troponin Release in Patients Suspected of Having CAD

We confirmed the existence of basal transcardiac troponin release, which had never been detected by the conventional troponin assay. The basal troponin release was shown to be correlated with peripheral serum levels of troponin. Furthermore, we originally identified factors that are significantly associated with elevated transcardiac troponin release using univariate and multivariate logistic regression analysis, whereas the earlier studies were based on only peripheral troponin revels [4], which might be influenced by skeletal muscle or renal function. These results may explain the minimal troponin elevation observed by this sensitive assay in stable CAD; this elevation was confirmed as one of prognostic factors of CAD, reflecting chronic myocardial injury [6], [14]. In a recent study, Turer et al. successfully showed that troponin was released due to myocardial ischemia induced by rapid atrial pacing [15].

### Transcardiac Troponin Release Before and After the ACh Test

Cardiac troponins exist in a structural form with tropomyosin and actin and in a free cytosolic pool [16]. Although a controversial view, reversible ischemia may induce changes in the permeability of the myocardial membrane and subsequent release of troponin from the cytosolic pool [17]. The ACh-induced transcardiac troponin release detected in the present study may reflect a part of cardiac ischemia that does not involve myocyte necrosis-induced troponin elevation in the absence of CAD. Transcardiac troponin release was increased within 1 minute after onset of ischemia, which may be earlier than expected. This observed earlier release may be due to the earlier detection of troponin elevation from cytosolic pool by means of coronary sinus sampling. Recent guidelines [18] note that 15% of patients with non ST-elevation acute coronary syndrome have normal coronary arteries or non-obstructive lesions. The results of the present study may partly explain the troponin elevation observed in such patients.

We used the ACh-test to provoke myocardial ischemia because we intended to exclude exercise-induced myocardial stretch, increased production of oxidative radicals, altered acid–base balance, or double product-dependent myocardial damage [19]. Although troponin elevation in the setting of exercise-induced ischemia has been reported [20], this observed release may be due to troponin elevation due to the exercise of skeletal muscles and not to myocardial ischemia [19]. In view of the diagnosis of vasospastic angina and the objective confirmation of spasm-induced myocardial ischemia, measurement of troponin levels during the ACh test may have a supplemental role for the diagnosis of vasospastic angina as well as the measurement of myocardial lactate consumption [5].

We used a highly sensitive assay from Roche Diagnostics. This assay has been commonly used in recently reported large observational studies [2], [6], [14].

### Limitations

The study population was relatively small because of the invasive nature of coronary sinus sampling. Although transcardiac troponin production was comparable between patients with and without hypertension or DM, the small number of patients might have caused a lack of statistically significant difference in this analysis. We assessed the “basal” transcardiac troponin release during angiography, but this method could not exclude silent ischemia because of the lack of functional studies such as the measurement of lactate in coronary sinus. The weak correlation between transcardiac troponin release and peripheral levels does not allow to draw definitive conclusions. The diagnostic value of transcardiac troponin release was insufficient for the purpose of CAD detection in clinical practice. The ACh test may cause total occlusion of the epicardial coronary artery with ST segment elevation, which is rarely observed in most CAD patients but which may occur in certain situations such as variant angina and acute coronary syndromes with non-obstructive coronary artery disease. The lack of follow-up data for the peripheral-vein levels of troponin just after the ACh-test or on the next day was a major limitation for all clinical applications. The appropriateness of the point of blood sampling from coronary sinus (1 minute after ACh-induced spasm) is uncertain. Serial sampling is necessary to determine the optimal time point. We did not have data on cardiovascular outcome, and the prognostic impact of transcardiac troponin release was not determined in the present study.

### Conclusions

Basal troponin release in the coronary circulation was observed in most patients with suspected CAD and coronary risk factor(s). Male sex, left ventricular hypertrophy determined by echocardiography, T-wave inversion, and CAD correlated with elevated troponin release in stable physical conditions. This sensitive assay could be used to detect significant increases in transcardiac troponin release provoked by coronary spasm-induced transient myocardial ischemia in patients without CAD.
